# Pubertal maturation and weight status are associated with dyslipidemia among children and adolescents in Northwest China

**DOI:** 10.1038/s41598-020-73507-0

**Published:** 2020-10-01

**Authors:** Juan Cao, Ling Zhang, Jing Li, Lijiao Sun, Shanghong Liu, Jianjun Zhang, Haiping Zhao

**Affiliations:** 1 Department of Child and Adolescent Health, School of Public Health and Management, Ningxia Medical University, Shengli Street, Yinchuan, 750004 Ningxia Hui Autonomous Region China; 2 Department of Epidemiology, Richard M. Fairbanks School of Public Health, Indiana University, 1050 Wishard Boulevard, RG5118, Indianapolis, IN 46202 USA

**Keywords:** Endocrinology, Health care

## Abstract

Dyslipidemia is one of major risk factors for cardiovascular disease. The early detection and treatment of dyslipidemia can reduce cardiovascular disease risk. A cross-sectional study was carried out in Ningxia, China to determine the prevalence of dyslipidemia and its association with body mass index (BMI) and pubertal stage. A total of 1783 students were selected from middle schools and high schools in September 2014 using stratified random cluster sampling. Serum triglyceride (TG), total cholesterol (TC), high-density lipoprotein cholesterol (HDL-C), and low-density lipoprotein cholesterol (LDL-C) were measured by using MOL-300 automatic biochemical analyzer with associated kits. The prevalence of adolescents with one abnormal serum lipid component was 43.2% and was significantly different across three pubertal stages (*p* < 0.0001). The abnormal rates of HDL-C and TG increased as the students maturated through the early, middle, and late stages of puberty (all *p* < 0.0001). Similar results were obtained when separate analyses were performed for boys and girls. In linear regression analysis, BMI was positively associated with serum levels of TC, LDL-C, and TG, but inversely associated with serum levels of HDL-C after the adjustment for age, sex, and race. In multivariable logistic regression analysis, obesity was associated with an increased risk of developing high TC, while pubertal maturation was associated with an elevated risk of experiencing low HDL-C and high TG (all *p* < 0.05). In conclusions, dyslipidemia is common in an adolescent population of Northwest China and its prevalence rates substantially vary with weight status and pubertal stage.

## Introduction

Dyslipidemia is one of the most common metabolic disorders and affects a substantial proportion of children and adolescents worldwide^1^. Numerous epidemiologic studies have shown that dyslipidemia is a strong risk factor with a pathological basis of atherosclerosis for coronary heart diseases^2,3^. The early onset of atherosclerosis has become more common in recent years, and atherosclerotic lesions have been detected in infants and even in fetuses^4,5^. It has been observed that the risk of cardiovascular disease (CVD) increased by 13% per 1 mmol/L triglyceride (TG) increment^6^. A report from the American Heart Association indicated that an average of one death every 39 s was caused by CVD^7^. The China’s Health and Family Planning Statistical Yearbook 2014^8^ showed that CVD accounted for 44.8% and 41.9% of all-cause mortality in rural and urban areas of China, respectively.

Rapid socioeconomic development during the last few decades in China has exerted a substantial impact on lifestyle factors (e.g. increased intake of fat and meats and reduced levels of physical activity) among Chinese residents. The Chinese Students’ Constitution and Health Survey showed that obesity prevalence in school-aged children and adolescents increased from 0.03% to 4.3% in 1985–2010^9^. Dyslipidemia prevalence rates have simultaneously increased in response to these changes in lifestyle factors and weight status^10^. A meta-analysis of a nationally representative sample of 129,426 children and adolescents aged 7–18 years in China revealed that dyslipidemia prevalence rate was 31.6% in this population^11^. Cross-sectional studies found that overweight and obesity were positively associated with hyperlipidemia prevalence in diverse pediatric populations^12,13^^.^ Therefore, it is biologically plausible that some epidemiological studies have reported that overweight in adolescence was a good predictor of CVD risk in adulthood. For example, the Third Harvard Growth Survey has found that overweight in adolescence was associated with a more than twofold increased risk of developing coronary heart disease in adulthood after a follow-up of 55 years, and this positive association was independent of adult weight^14^. Conversely, the Third National Health and Nutrition Examination Survey in the United States has shown that a decline of abnormal blood lipid profiles in childhood could reduce the risk of CVD and its morbidity and mortality in adulthood^15^. Furthermore, it has been suggested that the screening of dyslipidemia could be confined to young children as adolescents with dyslipidemia experienced a significantly elevated risk of developing high carotid artery intima-media thickness (an established risk factor for CVD) in adulthood^16^. Taken together, the findings of previous studies suggest that identification and prevention of dyslipidemia in children and adolescents can offer a novel and effective strategy for preventing CVD in adults.

Ningxia is an economically underdeveloped region of northwest China. In recent years, some studies have found that the prevalence rates of overweight and metabolic syndrome have considerably increased among children and adolescents residing in this area^17^. However, little is known about the blood lipid profiles of children and adolescents in different pubertal stages and their associations with body mass index (BMI). Blood lipid levels may vary across pubertal stages as hormonal changes during sexual maturation render children and adolescents (especially those with unhealthy dietary habits) vulnerable to the development of dyslipidemia^18–20^. Previous studies have revealed that serum levels of testosterone and leptin increased in girls during pubertal development^18,19^. It has been also reported that insulin resistance started to rise early in puberty^20^. Furthermore, increased levels of testosterone and leptin and onset of insulin resistance have been associated with adiposity in children and adolescents^18–20^. Therefore, the present study was sought to investigate the associations of serum lipid levels with puberty stage and adiposity among a large number of school-aged children in Ningxia.

## Results

Table 1 showed that serum TC and LDL-C levels were significantly higher in female children than male children. The levels of TC and LDL-C were elevated with increasing BMI. Blood lipid profiles were significantly different across three pubertal stages. There was a significantly gradient decline in mean values of LDL-C, HDL-C, and TC from early adolescence to late adolescence. Conversely, the increasing trend was observed for TG levels, with mean values being the lowest in early adolescence, intermediate in middle adolescence, and the highest in late adolescence.

**Table 1:** Blood lipid concentrations (mean ± SD, mg/dL) among children and adolescents in Ningxia, China, 2014.

		N	TC	LDL-C	HDL-C	TG
All subjects		1783	134.7 ± 23.1	62.4 ± 20.1	43.7 ± 12.1	103.1 ± 49.2
**Sex**	Male	881	132.5 ± 23.0	60.7 ± 20.4	43.6 ± 13.3	101.6 ± 49.7
	Female	902	136.4 ± 22.6	63.6 ± 19.4	43.6 ± 10.9	105.2 ± 49.0
		*p*	0.005	0.015	0.885	0.151
**Weight status**	Normal	1535	132.8 ± 21.9	61.2 ± 19.1	43.6 ± 11.7	101.1 ± 47.5
	Overweight	192	143.1 ± 26.3	67.5 ± 23.4	42.8 ± 12.1	118.6 ± 59.0
	Obesity	56	148.2 ± 26.7	70.9 ± 25.4	45.7 ± 20.2	114.3 ± 53.1
		*p*	< 0.0001	< 0.0001	0.363	< 0.0001
**Pubertal stages**	Early	950	138.0 ± 23.5	65.8 ± 19.6	45.9 ± 12.3	97.1 ± 47.5
	Middle	591	129.8 ± 22.2	57.9 ± 18.9	41.0 ± 11.7	108.6 ± 51.2
	Late	242	132.6 ± 19.7	59.0 ± 21.2	41.4 ± 10.4	114.9 ± 48.3
		*p*	< 0.0001	< 0.0001	< 0.0001	< 0.0001

*TC* total cholesterol, *LDL-C* low density lipoprotein cholesterol, *HDL-C* high density lipoprotein cholesterol, *TG* triglyceride.

Abnormal prevalence rates of serum TC, LDL-C, HDL-C, and TG among all study children were 1.3%, 0.4%, 37.4% and 16.4%, respectively (Table 2). Dyslipidemia prevalence rates, especially those of HDL-C and TG, significantly increased from early adolescence to late adolescence (*p* < 0.05). No significant or apparent differences were observed for TC and LDL-C. The prevalence rates of abnormal levels of TC, LDL-C and TG were significantly higher in overweight and obese children than those of normal weight children.

**Table 2:** Prevalence of dyslipidemia among children and adolescents in Ningxia, China, 2014.

		N	TC n, %	LDL-C n, %	HDL-C n, %	TG n, %
All subjects		1783	23, 1.3	8, 0.4	667, 37.4	293, 16.4
**Sex**	Boys	881	13, 1.5	6, 0.7	348, 39.5	146, 16.6
	Girls	902	10, 1.1	2, 0.2	319, 35.4	147, 16.3
		*P*	0.49	0.15	0.071	0.88
**Weight status**	Normal	1535	9, 0.6	4, 0.3	585, 38.1	243, 15.5
	Overweight	192	9, 4.7	2, 1.0	84, 43.8	37, 23.1
	Obesity	56	5, 8.9	2, 3.6	24, 42.9	13, 23.2
		*P*	< 0.0001	0.007	0.26	0.018
**Pubertal stage**	Early	950	17, 1.8	8, 0.8	285, 30.0	118, 12.4
	Middle	591	5, 0.8	0, 0.0	267, 45.2	120, 20.3
	Late	242	1, 0.4	0, 0.0	115, 47.5	55, 22.7
		*P*	0.15	0.044	< 0.0001	< 0.0001

*TC* total cholesterol, *LDL-C* low density lipoprotein cholesterol, *HDL-C* high density lipoprotein cholesterol, *TG* triglyceride.

The numbers and proportion of adolescents with dyslipidemia are presented in Table 3. One or two abnormal lipid components significantly increased across three pubertal stages (*p* < 0.05). The prevalence rates of children with one and two abnormal lipid components were 43.2% and 12.2%, respectively. Of the 1783 children and adolescents examined, only 6 developed three abnormal lipid components. Similar results were obtained for boys and girls; the prevalence rates of one abnormal lipid component were 45.2% for boys and 41.2% for girls.

**Table 3:** Numbers and prevalence of abnormal lipid components by adolescent stage in Ningxia, China, 2014.

	One abnormal component	Two abnormal components	Three abnormal components
	N (%)	N (%)	N (%)
**Total**	770 (43.2)	217 (12.2)	6 (0.34)
Early adolescence	331 (34.8)	95 (10.0)	4 (0.42)
Middle adolescence	308 (52.1)	83 (14.0)	1 (0.17)
Late adolescence	131 (54.1)	39 (16.1)	1 (0.41)
*p*	< 0.0001	0.008	0.62
**Boys**	398 (45.2)	113 (12.8)	4 (0.45)
Early adolescence	157 (35.1)	48 (10.7)	3 (0.67)
Middle adolescence	159 (52.8)	41 (13.6)	0 (0.0)
Late adolescence	82 (61.7)	24 (18.0)	1 (0.75)
*p*	< 0.0001	0.076	0.35
**Girls**	372 (41.2)	104 (11.5)	2 (0.22)
Early adolescence	174 (34.6)	47 (9.3)	1 (0.20)
Middle adolescence	149 (51.4)	42 (14.5)	1 (0.34)
Late adolescence	49 (45.0)	15 (13.8)	0 (0.0)
*P*	< 0.0001	0.068	0.797

After adjustment for age, sex, and race, BMI was positively associated with serum levels of TC, LDL-C, and TG but inversely associated with serum levels of HDL-C. Similar results were obtained when separate analyses were performed for boys and girls (all *p* < 0.05) (Table 4). Table 5 showed that children at late adolescence exhibited more than twofold risk of developing abnormal levels of HDL-C and TG than those at early adolescence after adjustment for sex and weight status. Overweight and obese children had a remarkably increased risk of being detected with abnormal levels of TC, which was independent of pubertal stage and sex. All these associations remained significance after additional adjustment for parent education and race (all *p* < 0.05) (Table 5).

**Table 4:** Linear regression between BMI and blood lipid concentrations among 1783 children and adolescents in Ningxia, China, 2014.

		TC (mg/dL)*	LDL-C (mg/dL)*	HDL-C (mg/dL)*	TG (mg/dL)*
**All subjects**	*β*	0.508	0.795	− 0.648	1.235
	95% *CI*	0.260, 0.755	0.515, 0.975	− 1.142, − 0.034	1.001, 1.517
	*P*	< 0.0001	0.003	0.010	< 0.0001
**Boys**	*Β*	0.584	0.757	− 0.697	1.233
	95% *CI*	0.217, 0.951	0.348, 0.966	− 1.335, − 0.058	1.048, 1.619
	*P*	0.002	< 0.0001	0.032	< 0.0001
**Girls**	*Β*	0.449	0.523	− 0.143	1.290
	95% *CI*	0.113, 0.785	0.247, 0.798	− 0.839, − 0.447	1.094, 1.639
	*p*	< 0.0001	< 0.0001	< 0.0001	< 0.0001

*TC* total cholesterol, *LDL-C* low-density lipoprotein cholesterol, *HDL-C* high-density lipoprotein cholesterol, *TG* triglycerides, *CI* confidence interval.

*Adjustment for age, sex, and race.

**Table 5:** Associations of pubertal stage, sex, and BMI with risk of dyslipidemia among children and adolescents in Ningxia, China, 2014.

Variables	N	OR (95% CI)^†^			
		TC	LDL-C	HDL-C	TG
**Model 1**					
**Pubertal stage**^‡^					
Early adolescence Middle adolescence Late adolescence	950 591 242	1.00 0.55 (0.20, 1.52) 0.25 (0.03, 1.93)	– – –	1.00 **1.99 (1.59, 2.47)** **2.19 (1.62, 2.95)**	1.00 **1.84 (1.39, 2.44)** **2.13 (1.49, 3.05)**
**Sex**					
Girls Boys	902 881	1.00 0.19 (0.50, 2.81)	1.00 3.11 (0.62, 15.7)	1.00 1.14 (0.93, 1.39)	1.00 1.05 (0.81, 1.35)
**Weight status**					
Normal weight Overweight Obesity	1535 192 56	1.00 **7.88 (3.07, 20.2)** **12.1 (3.57, 41.3)**	1.00 1.55 (0.02, 13.5) **8.38 (1.55, 45.2)**	1.00 1.36 (0.99, 1.86) 1.35 (0.77, 2.39)	1.00 **1.72 (1.15, 2.55)** 1.84 (0.97, 3.50)
**Model 2**					
**Pubertal stage**					
Early adolescence Middle adolescence Late adolescence	950 591 242	1.00 0.48 (0.13, 1.73) 0.44 (0.06, 3.46)	**–** **–** **–**	1.00 **2.14 (1.68, 2.72)** **2.70 (1.91, 3.81)**	1.00 **2.13 (1.57, 2.91)** **3.14 (2.09, 4.71)**
**Sex**					
Girls Boys	902 881	1.00 1.12 (0.40, 3.16)	1.00 1.94 (0.35, 10.77)	1.00 1.14 (0.91, 1.42)	1.00 0.91 (0.68, 1.20)
**Weight status**					
Normal weight Overweight Obesity	1535 192 56	1.00 **4.12 (1.23, 13.83)** **6.49 (1.35, 31.23)**	1.00 2.09 (0.23, 19.50) 7.43 (0.79, 69.10)	1.00 1.44 (0.99, 2.10) 1.31 (0.69, 2.46)	1.00 0.80 (0.36, 1.73) 1.46 (0.60, 3.53)

Dyslipidemia is defined as: TC ≥ 200 mg/dL, TG ≥ 150 mg/dL, LDL-C ≥ 130 mg/dL, and HDL-C ≤ 40 mg/dL. ORs shown in bold font are statistically significant at *p* < 0.05.

*OR* odds ratio, *CI* confidence interval, *TC* total cholesterol, *LDL-C* low-density lipoprotein cholesterol, *HDL-C* high-density lipoprotein cholesterol, *TG* triglycerides.

^†^Model 1, three variables were mutually adjusted in logistic regression analysis; Model 2, parental education and race were additionally adjusted.

^‡^OR (95% CI) could not be estimated for middle adolescence and late adolescence groups as all eight children with abnormal LDL-C levels were in early adolescence group (reference).

## Discussion

The present study revealed that 43.2% of students in middle schools and high schools in two cities of Northwest China have developed at least one abnormal lipid component. The main types of dyslipidemia were low levels of serum HDL-C and high levels of serum TG. The abnormal rates of serum HDL-C and TG increased as the students maturated through the early, middle, and late stages of puberty. BMI was positively correlated with serum TC, LDL-C, and TG but inversely correlated with HDL-C.

A cohort study carried out in Denmark reported that plasma concentrations of TC, LDL-C, and HDL-C were higher in younger children and adolescents than in older ones, but opposite results were obtained for TG^21^. Conversely, our study showed that the concentrations of serum TC, LDL-C, and HDL-C among children at late adolescence were significantly lower than those at early adolescence, which was consistent with the results of some previous studies^22,23^. This downward trend was probably due to hormonal changes during pubertal maturation^24^. The present study also found that a gradient increase in serum TG concentrations occurred from early adolescence to late adolescence. This change has been reported in other studies^25,26^. Alterations in blood lipid profiles during puberty may be ascribed to various factors, including reproductive development (e.g. spermatorrhea in boys and menarche in girls), dietary intake of nutrients, and body fatness. Such alterations may be mediated through a gradual increase in serum testosterone and leptin levels and an apparent emergence of insulin resistance across sexual maturation. An almost linear decrease in HDL-C levels were identified among both black and white boys throughout pubertal development in parallel with rapid increase in free testosterone levels^27^.

The prevalence rates of dyslipidemia vary among countries across the world. An analysis of the National Health and Nutrition Examination Survey data showed that the prevalence rates of dyslipidemia among American children and adolescents were 7.8% for TC and 12.8% for HDL-C in 2011–2012^4^. Dyslipidemia was present in 34.3% of Eastern Iranian adolescents^28^. Our study found that the prevalence rate of dyslipidemia was 37.4% for HDL-C and 16.4% for TG among children and adolescents in a Northwest area of China. These differences in dyslipidemia between populations may be attributable to their differences in dietary habits, physical activity, and genetic predisposition. However, caution should be exercised in comparison of dyslipidemia rates between those studies because they used different cutoff points for defining abnormal lipid components. In our study, dyslipidemia was defined with the normative cutoff points of the expert consensus on the prevention and treatment of dyslipidemia among Chinese children and adolescents^29^.

With the rapid economic development and improved living standards in the last few decades, China is facing a dual health burden of undernutrition among some rural residents and overnutrition among some urban residents. The Chinese National Survey on Students’ Constitution and Health reported that the prevalence rates of overweight and obesity have rapidly increased since early 1990s. In 2010, the age-adjusted prevalence of overweight and obesity was 11.1% and 8.1% in children and adolescents (aged 7–18 years), respectively^30^. The upward trends in prevalence of overweight and obesity might have occurred at least in part as a consequence of nutrition transition in China. Specifically, substantial changes in dietary composition have taken place in China. Diet has changed from foods high in carbohydrates and fiber to a diet high in total fat and meats among a considerable proportion of Chinese residents^31^. Per capita consumption of animal foods and fat increased, whereas that of carbohydrates decreased. At the same time, average levels of physical activity among Chinese people have been substantially reduced due to the popularity of private cars and improvement of public transportation systems^32^.

The present study showed that overweight and obese adolescents had higher levels of TC, LDL-C, and TG, compared with those with normal BMI. In addition, BMI was positively correlated with TC, LDL-C, and TG but inversely correlated with HDL-C after adjustment for age, sex, and race. Several studies have found that obese children had higher levels of LDL-C, TG, and TC but lower levels of HDL-C than those with normal weight^33^ and that BMI was positively associated with hyperlipidemia^34^. A Chinese study reported that hyperlipidemia risk is 1.5 times higher in overweight children and 1.8 times higher in the obese children than in the normal-weight children^35^. The results of our study and those previous studies are in agreement with reported positive associations of high animal-fat diet, sedentary lifestyle, and obesity with adverse blood lipid profiles in various populations^36,37^. With regard to potential biological mechanisms for the adverse influence of obesity on blood lipid profiles, it has been put forward that obesity modulates blood lipid concentrations by impairing the endothelial functions of the blood vessels, reducing insulin resistance, and enhancing oxidative stress, which leads to an elevated risk of developing dyslipidemia and CVD^38,39^.

In the present study, the abnormal rates of serum HDL-C and TG increased monotonically across the three stages of puberty, which reflects our observation of decrease in HDL-C levels and increase in TG levels over the course of pubertal development. It is possible that these trends in HDL-C and TG is primarily driven by changes in sex hormones during this phase of rapid sexual maturation^27^. We are not able to investigate this hypothesis as serum testosterone and estrogen were not measured in our study. The abnormal rate of HDL-C was 37.4% and that of TG was 16.4% among school-aged children enrolled to the present study in 2014, which was higher than the abnormal rate of HDL-C (7.4%) and TG (11.9%) in a study of Chinese adults evaluated in 2002^40^. These differences are in part attributable to more stringent criteria for defining abnormal levels of HDL-C (< 35 mg/dL) and hypertriglyceridemia (≥ 150 mg/dL) in that Chinese adult study, age-related differences in lipid metabolism, and the aforementioned nutrition transition and declining physical activity levels that had occurred between 2002 and 2014. Regardless of the potential reasons for the above differences, our observation of decrease in HDL-C levels and increase in TG levels with the sexual maturation of children suggest that it is critical and warranted to educate children and adolescents to lead a healthy lifestyle characterized by low intake of meat, fat, and sugared beverages, high intake of vegetables and fruits, and sufficient amounts of physical activity to reduce the risk of developing dyslipidemia and subsequent CVD.

The advantages of the present study include a large randomly selected sample of students who are well representative of children and adolescents in Ningxia with regard to age, sex, and race/ethnicity, which is supported by comparing the data of our study subjects with data obtained from the 2014 National Survey on Students’ Constitution and Health in Ningxia^41^. Data analyzed in our study were collected from children and adolescents residing in an underdeveloped and understudied region of China. This region has a high proportion of Hui ethnic minority people whose dietary habits (characterized by high intake of mutton and avoidance of pork) are different from those of people in other parts of China. Therefore, the findings of the present study have accentuated the consistency of the association between adiposity and blood lipid levels, which has been observed in previous studies. Furthermore, our study is among the first to reveal that pubertal stage and BMI were independently associated with risk of dyslipidemia in an Asian pediatric population.

The limitations of the present study need to be considered in the interpretation of obtained results. The cross-sectional nature of our study design prevents us from drawing causal inference for the associations of pubertal maturation and weight status with dyslipidemia. Both BMI and blood lipids were measured at one point in time. We are thus not allowed to investigate the longitudinal influence of BMI on blood lipid profiles. In our study, pubertal stages were classified by age, rather than by clinical assessment, which failed to consider the fact that puberty generally starts earlier for girls. Another limitation of our study is lack of data on lifestyle factors that may also affect levels of blood lipids (e.g. diet, alcohol, smoking, physical activity)^42,43^. Therefore, residual confounding might have somewhat distorted our obtained results.

In conclusion, the present study demonstrated that dyslipidemia is common and its prevalence gradually increases across three pubertal stages among children and adolescents in Ningxia, China. The main types of dyslipidemia were low levels of HDL-C and high levels of TG. Our study has generated highly needed data on blood lipid profiles for school-aged children in two cities of Northwest China. Such data are critical and valuable for public health agencies to formulate intervention strategies for preventing dyslipidemia and subsequent CVD, including promotion of physical activity, healthy diet (low in fat, red meats, and refined sugar and high in vegetables and fruits), and health education^44^.

## Materials and methods

### Study population

Data analyzed were obtained from the Chinese Students’ Constitution and Health Survey in Ningxia. Stratified random cluster sampling was used for subject recruitment. Two of five regions in Ningxia (Yinchuan City and Wuzhong City) were selected as survey sites. Seven schools, including four middle schools and three high schools, were randomly selected from all middle and high schools of these two cities. Three classes from each grade were chosen from each of the seven schools selected. All students in selected classes were invited to participate in the survey. Students in grades 9 and 12, who would graduate from middle schools and high schools, respectively, were excluded due to potential low response rates. A total of 1890 students aged 10–18 years were enrolled to the survey. Of these 1783 (including 881 males and 902 females) successfully completed the survey and were included as study subjects in the present study. The survey protocol was approved by the Medical Ethical Committee of Ningxia Medical University, both the students and their parents or guardians provided written informed consent before entry into this study according to the regulations, and the study was performed according to the Declaration of Helsinki.

### Anthropometric measurements

Height without shoes (cm) and weight (kg) with light clothes were measured using a portable weighing scale with height rod (TXRGZB-200-RT). For each of selected children and adolescents, both height and weight were determined twice and the mean values of two measurements were used in data analysis. BMI was calculated as kg/m^2^. Overweight and obesity were defined as a BMI at or above the 85th and 95th percentiles for children and adolescents of the same age and sex, respectively^45^.

### Laboratory analysis

Blood samples were collected from the antecubital vein between 7 and 8 am after 12 h of fasting. Samples were collected in coagulation tubes. One set of blood samples from each subject was collected in lithium heparin vacuum tubes. Serum was separated by centrifugation at 3000 r for 15 min at 4 ℃ within 2 h on the survey site. Serum was transferred to separate tubes, labeled, stored in ice, and then sent to the laboratory immediately. The samples were frozen at − 80℃ until analysis. Serum TG, total cholesterol (TC), high-density lipoprotein cholesterol (HDL-C), and low-density lipoprotein cholesterol (LDL-C) were determined with a MOL-300 automatic biochemical analyzer (Li Kang Biological Medical Technology Holdings Group, Hong Kong), using glycerophosphate oxidase, peroxidase, 4-aminoantipyrine, and phenol method, cholesterol oxidase, peroxidase, 4-aminoantipyrine, and phenol method, antibody hindrance homogeneous method, and selective masking homogeneous method, respectively.

### Pubertal stage and abnormal blood lipids

Three pubertal stages were defined by using the criteria described in Nelson Textbook of Pediatrics^46^: early adolescence (aged 10–13 years), middle adolescence (aged 14–16 years), and late adolescence (aged 17–20 years). The expert consensus on the prevention and treatment of dyslipidemia among Chinese children and adolescents were used to define abnormal lipid components: TC ≥ 200 mg/dL (5.18 mmol/L), TG ≥ 150 mg/dL (1.70 mmol/L), LDL-C ≥ 130 mg/dL (3.37 mmol/L), and HDL-C ≤ 40 mg/dL (1.04 mmol/L)^29^. Children with one or more abnormal components of blood lipids are considered to have dyslipidemia.

### Statistical analysis

Continuous variables were expressed as mean ± standard deviation (SD). Continuous and categorical variables were analyzed by analysis of variance and chi-square test, respectively. Linear regression analysis and logistic regression analysis were performed to evaluate the associations of BMI, pubertal stage, and/or sex with serum blood lipids. A *p*-value of < 0.05 was considered statistically significant. All statistical analysis was carried out with SPSS 17.0 (Microsoft, Chicago IL, USA).

## Data Availability

The datasets generated during and/or analyzed during the current study are not publicly available due to the confidentiality of data but are available from the corresponding author on reasonable request.

## Abbreviations

BMI: Body mass index
HDL-C: High density lipoprotein cholesterol
LDL-C: Low density lipoprotein cholesterol
TG: Triglyceride
TC: Total cholesterol
CVD: Cardiovascular disease
